# Chronic stress and antidepressant treatment alter purine metabolism and beta oxidation within mouse brain and serum

**DOI:** 10.1038/s41598-020-75114-5

**Published:** 2020-10-22

**Authors:** Peter J. Hamilton, Emily Y. Chen, Vladimir Tolstikov, Catherine J. Peña, Joseph A. Picone, Punit Shah, Kiki Panagopoulos, Ana N. Strat, Deena M. Walker, Zachary S. Lorsch, Hannah L. Robinson, Nicholas L. Mervosh, Drew D. Kiraly, Rangaprasad Sarangarajan, Niven R. Narain, Michael A. Kiebish, Eric J. Nestler

**Affiliations:** 1 Nash Family Department of Neuroscience and Friedman Brain Institute, Icahn School of Medicine at Mount Sinai, One Gustave L Levy Place, New York, NY 10029 USA; 2 Department of Anatomy and Neurobiology, Virginia Commonwealth University, Richmond, VA 23298 USA; 3 BERG LLC, 500 Old Connecticut Path, Framingham, MA 01701 USA

**Keywords:** Stress and resilience, Molecular neuroscience

## Abstract

Major depressive disorder (MDD) is a complex condition with unclear pathophysiology. Molecular disruptions within limbic brain regions and the periphery contribute to depression symptomatology and a more complete understanding the diversity of molecular changes that occur in these tissues may guide the development of more efficacious antidepressant treatments. Here, we utilized a mouse chronic social stress model for the study of MDD and performed metabolomic, lipidomic, and proteomic profiling on serum plus several brain regions (ventral hippocampus, nucleus accumbens, and medial prefrontal cortex) of susceptible, resilient, and unstressed control mice. To identify how commonly used tricyclic antidepressants impact the molecular composition in these tissues, we treated stress-exposed mice with imipramine and repeated our multi-OMIC analyses. Proteomic analysis identified three serum proteins reduced in susceptible animals; lipidomic analysis detected differences in lipid species between resilient and susceptible animals in serum and brain; and metabolomic analysis revealed dysfunction of purine metabolism, beta oxidation, and antioxidants, which were differentially associated with stress susceptibility vs resilience by brain region. Antidepressant treatment ameliorated stress-induced behavioral abnormalities and affected key metabolites within outlined networks, most dramatically in the ventral hippocampus. This work presents a resource for chronic social stress-induced, tissue-specific changes in proteins, lipids, and metabolites and illuminates how molecular dysfunctions contribute to individual differences in stress sensitivity.

## Introduction

Major Depressive Disorder (MDD), a complex, heterogeneous syndrome, is the leading cause of disability worldwide. The symptoms of MDD range from emotional and cognitive impairments as well as systemic dysfunctions. These diverse symptoms suggest the dysregulation of multiple brain regions and peripheral tissues, and there is evidence for brain region-specific disruptions in MDD^1^. Tricyclic antidepressants (TCAs) initially inhibit serotonin and/or norepinephrine reuptake, but the brain signaling networks affected upon their chronic administration—required for therapeutic efficacy—remain insufficiently understood.

Here we utilize an ethologically-validated mouse model for the study of depression called chronic social defeat stress (CSDS), where mice are exposed chronically to a social stress, which induces a range of MDD-like behavioral and molecular changes in a subset (~ 50%) of animals, referred to as susceptible^2,3^. These defects are ameliorated by chronic antidepressant treatment^2,4,5^. The remainder of the stress-exposed population does not display most of these behavioral abnormalities and are referred to as resilient^3,4^. This divergence in vulnerability to stress is observed within human populations^6^.

In the present study, we employ metabolomic, lipidomic, and proteomic analyses of the ventral hippocampus (vHipp), nucleus accumbens (NAc), and medial prefrontal cortex (mPFC)—all implicated in MDD—and serum samples from susceptible, resilient, and control (stress naïve) mice in order to comprehensively quantify the changes that occur within these tissues in response to CSDS. We interrogated these brain regions specifically, since each is spatially distinct and contributes uniquely to the limbic system, a circuit of inter-connected brain regions that has been implicated across multiple levels of analysis in the context of depression in both human and animal studies^4,7,8^. We hypothesized that molecular profiles within these limbic regions would be distinct between resilient and susceptible animals, and that identifying which molecules and pathways are most different would shed light onto the factors responsible for behavioral stress-responses.

Using this approach, we discover that many of the molecules affected by CSDS are involved in the molecular pathways of nucleotide metabolism, fatty acid beta oxidation, and antioxidant function. These pathways are differentially associated with susceptibility vs resilience depending on the brain region involved. We also analyzed the effect of chronic administration of imipramine, a standard tricyclic antidepressant, on these multi-OMIC endpoints. We observe that many of the same pathways are affected by imipramine treatment, further evidence that activity of these pathways contributes to stress responses. Together, this work provides a rich dataset to explore the tissue-specific, molecular mechanisms that differentiate stress resilient and stress susceptible animals, and outlines strongly-affected protein, lipid, and metabolite pathways that present promising targets for antidepressant drug discovery or biomarker efforts.

## Methods and materials

### Animals

Adult male 7–8 week old C57BL/6 J mice and 6-month old CD1 retired male breeders (CD1 aggressors) were housed at 22–25 °C in a 12-h light/dark cycle and provided food and water ad libitum. All methods were conducted in accordance with the IACUC regulations at Mount Sinai (LA12-00051) and Virginia Commonwealth University (AD10002174). All experiments were approved by the IACUC at these institutions and were performed in accordance with relevant guidelines and regulations.

### Chronic social defeat stress and behavioral assays

We utilized an established CSDS protocol as described previously^2,3,9^. C57BL/6 J mice were exposed for ten consecutive days to a novel aggressive CD1 retired breeder for 10 min and were then separated from the aggressor by a perforated divider to maintain 24 h sensory contact. Each day the test mouse encountered a novel CD1 aggressor mouse. Mice were tested for social interaction (SI) 24 h after the last social defeat by first allowing 2.5 min for the test mouse to explore an arena containing a plexiglass wire mesh cage centered against one wall of the arena (target absent). In the second 2.5 min test, the same test mouse was returned to the arena with a novel CD1 mouse contained in the plexiglass and wire cage (target present). Across all SI tests for a given experiment, the same unfamiliar CD1 (i.e. not used in defeats) target mouse was used to provide consistent social interaction for our test mice. Based on the social interaction ratio, defined as time spent in the ‘interaction zone’ with target present divided by the time spent with target absent, mice were characterized as susceptible (SI ratio < 1) or resilient (SI ratio > 1). The SI ratio of 1 is a commonly used cut-off to discriminate resilient and susceptible animals^10–12^. Control mice were housed identically, yet never came in physical or sensory contact with a CD1 aggressor.

For antidepressant experiments, control, resilient, and susceptible populations were single housed and treated twice-daily with intraperitoneal (IP) injections of saline or imipramine (10 mg/kg) for 14 consecutive days after the SI test^5^. Treatment-induced changes in MDD-like behaviors were quantified by re-analyzing social interaction behaviors and performing elevated plus maze analysis. For the latter, mice were tested in a standard maze for 10 min, monitored by Ethovision XT as described previously^13^. Time in the open arms of the plus maze was quantified and expressed as a percent of total time.

### Tissue preparation

**Figure 1:**
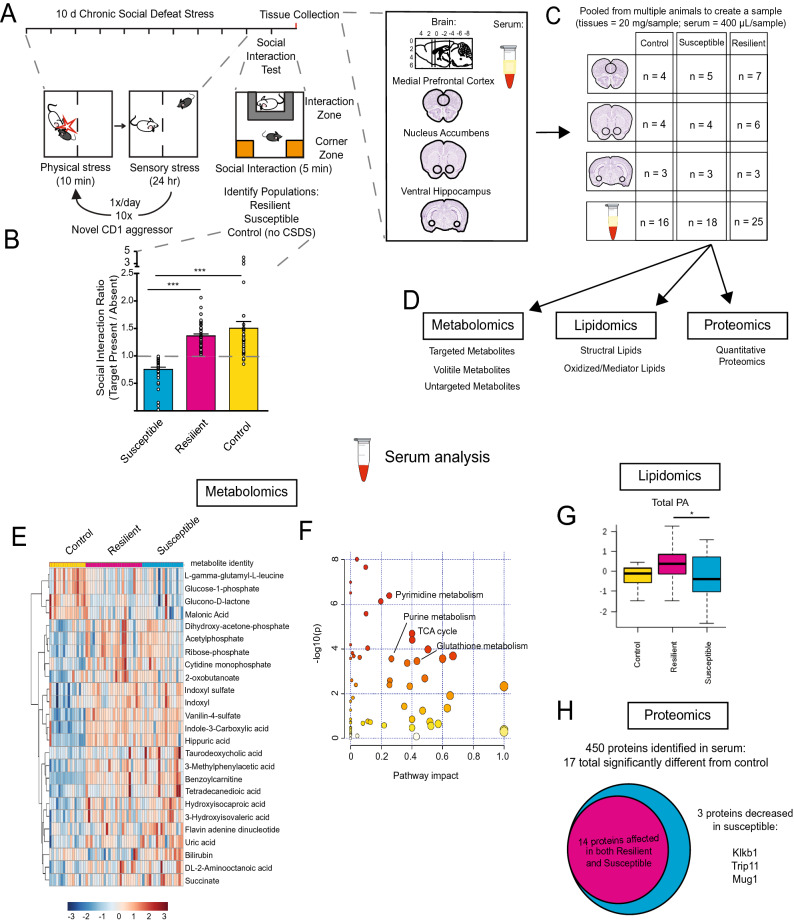
Study overview and metabolomic, lipidomic, and proteomic analysis of serum from resilient and susceptible populations of chronically stressed mice. (**A**) Graphical illustration of workflow for chronic social defeat stress (CSDS) to differentiate mice into susceptible and resilient populations. All tissues harvested for analysis are displayed. Coronal brain images credit: Allen Institute. (**B**) Social interaction (SI) data from all mice, tested 24 h after the last CSDS bout. SI ratio is quantified for each mouse, with resilience as an SI ratio > 1 and susceptibility as an SI ratio < 1. Susceptible (n = 37), Resilient (n = 50), and Control (n = 33) (*F*_2,117_ = 32.82; ***p < 0.0001; one-way ANOVA followed by Bonferroni post-test). (**C**) To generate sufficient material for parallel analyses, tissues were pooled by SI ratio to achieve 20 mg/sample for brain tissues and 400 μL/sample for serum. Reported values are the sample numbers for each condition. A single mPFC sample consists of the pooled tissue of seven animals, a single NAc sample consists of the bilateral punches of eight animals, a single vHipp sample consists of the bilateral punches of 11 animals, and a single serum sample is pooled from two animals. (**D**) From this pooled sample, all processing and analysis occurred in parallel. (**E**) Heatmap of top 25 affected serum metabolites shows differences in metabolite levels, localized to experimental groups. (**F**) Pathway analysis of changed metabolites in the serum reveals purine and pyrimidine metabolism, the tricarboxylic acid cycle (TCA cycle), and antioxidant function, among other functions, as significantly affected in the serum of these chronically-stressed mice. Metabolites comprising “Pyrimidine metabolism” network are: Glutamine; Carbamoyl phosphate; Orotidine 5′-phosphate; Uridine; CMP; Cytidine; Deoxycytidine; Deoxyuridine; Thymidine; Thymine; N-Carbamoyl-L-aspartate; Orotate; Uracil; 3-Aminoisobutyrate. “Purine metabolism” network: Xanthine; D-Ribose 5-phosphate; L-Glutamine; 1-(5′-Phosphoribosyl)-5-amino-4-imidazolecarboxamide; AMP; IMP; Adenosine; dAMP; Deoxyadenosine; Deoxyinosine; Xanthosine; Hypoxanthine; Inosine; Guanines; Allantoate; Guanosine; Adenine; Urate; Aminoimidazole ribotide; Urea; Allantoin. “TCA cycle” network: 2-Oxoglutarate; Succinate; Isocitrate; Malate; cis-Aconitate; Citrate; Pyruvate; Fumarate. “Glutathione metabolism” network: Glutathione; NADP + ; Glutathione disulfide; Glycine; L-Glutamate; L-Cysteine; 5-Oxoproline; L-Ornithine; Spermidine. (**G**) Lipidomic serum analysis reveals total circulating levels of phosphatidic acid (PA) increased in animals resilient to CSDS relative to susceptible animals (*F*_2,58_ = 3.80; *p < 0.05; one-way ANOVA followed by Fisher’s LSD comparing resilient and susceptible). (**H**) Proteomic analysis of serum: a total of 450 proteins were detected, with 17 proteins identified as significantly different from undefeated control animals. Of these 17, three proteins were significantly decreased solely in the susceptible cohort: kallikrein B1 (Klkb1), murinoglobulin-1 (Mug1), and thyroid receptor-interacting protein 11 (Trip11). Images in E, F, and G were generated with MetaboAnalyst 4.0 (https://www.metaboanalyst.ca/).

Twenty-four hours after behavioral testing, vHipp, NAc, and mPFC tissue and serum were collected from consciously decapitated animals, immediately frozen, and stored at − 80 °C. Brains were sectioned in the coronal plane to 1 mm thickness in a brain matrix. Two 16-gauge punches (internal diameter 1.19 mm) were used to microdissect bilateral vHipp, two 14-gauge punches (internal diameter 1.6 mm) were used to isolate NAc bilaterally, and a single 12-gauge tissue punch (internal diameter 2.16 mm) was used to microdissect the mPFC. See Fig. 1A for size and targeting of the tissue punches. Tissue punches were pooled from between 7 and 11 animals depending on the mass of the brain region in order to create a single ~ 20 mg sample—a mass required to run metabolomics, lipidomics, and proteomics in parallel from the same sample. The bilateral vHipp punches of 11 animals were combined to create a single vHipp sample, bilateral NAc punches of eight animals were pooled to create a single NAc sample, and mPFC punches of seven animals were pooled to create a single mPFC sample. The exact number of samples per group is shown in Fig. 1C. The samples were combined in Omni homogenization bead tubes. The sera from two animals were pooled to create 400 µL samples. The aggregation of tissue and serum was performed to equalize SI ratios for samples in each group (resilient SI ratio: ~ 1.4; susceptible SI ratio: ~ 0.8; control SI ratio: ~ 1.35). From these pooled samples, all analyses were performed in parallel. Pooled samples were homogenized in water at 4 °C in an Omni Bead Ruptor 24 (Omni International, Tulsa, OK) and the protein content of each homogenate were determined via a bicinchoninic assay. Aliquots of 100 µg protein, 10 mg tissue weight, and 0.5 mg protein were separated for proteomics, metabolomics, and structural lipidomics analysis, respectively. Aliquots of pooled serum samples were likewise taken for proteomics, metabolomics, structural lipidomics, and mediator lipidomics analysis.

### Targeted and untargeted metabolomic analysis

Metabolomic analyses were performed using gas chromatography–mass spectrometry (GC/MS), reversed-phase liquid chromatography–mass spectrometry (RP-LC/MS), and hydrophilic interaction chromatography–liquid chromatography–tandem mass spectrometry (HILIC-LC/MS/MS)^14–17^. Metabolite extraction was achieved using a mixture of isopropanol:acetonitrile:water (3:3:2 v/v/v). Tissue samples were homogenized in an extraction mixture using Fisherbrand Model 120 Sonic Dismembrator. Extract analysis was performed using GC/MS, RP-LC/MS, and HILIC-LC/MS/MS protocols as described^14^. Quality control was performed using metabolite standards mixtures and pooled samples. The pooled QC sample was obtained by taking an aliquot of the same volume of all samples from the study. Supernatants of tissue and serum extracts were divided in three parts: 75 µL for GC-TOF–MS analysis, 75 µL for RP-LC/MS analysis, and 100 µL for HILIC-LC/MS/MS analysis*.* Collected raw data were manually inspected, merged, and imputed.

Specifically, metabolomics data were acquired using GC/MS, RP-LC/MS, and HILIC-LC–MS/MS. Recorded mass chromatograms were used for chromatography peak integration. Manual inspection of each mass chromatogram was performed to make sure no failure of separation unit and/or mass spectrometer occurred, and to ensure the acquired data were within the linear range of measurements. Peak areas of the identified metabolites were obtained using vendor’s integration software: ChromaTOF (LECO) and MultiQuant (Sciex). Three datasets containing integrated data for actual batch and all the QCs and pooled samples were merged in a single dataset (i.e. excel sheet) after normalization using external QCs (pooled control human plasma obtained from the UK Biobank, and injected after each 10 samples in a batch) and pooled batch samples was accomplished. Prior normalization missing values imputation was performed with the removal entities having more than 50% of missing or not detected data (N/D or below LOD). Duplicates removal followed with the hierarchy LC–MS/MS > LC–MS > GC–MS based on previously calculated CV for each duplicate detected with using different coupling. Further generalized log (glog) transformation and autoscaling were applied to stabilize the variability of the data. Negative values resulted for peak areas having raw values below 1 (arbitrary unit). Waaijenborg et al. provides additional information on our approaches for fusing metabolomics data from different platforms^18^.

Statistical analysis was performed with MetaboAnalyst 4.0^19^. Metabolite Set Enrichment Analysis (MSEA) was used to interrogate functional relations, which describes the correlation between compound concentration profiles and clinical outcomes.

### Structural lipidomic analysis

A cocktail of deuterium-labeled and odd chain phospholipid standards from diverse lipid classes was added to 25 µL of thawed serum or tissue homogenate with 0.5 mg protein as measured via a bicinchoninic assay. Standards were chosen so that they represented each lipid class and were at designated concentrations chosen to provide the most accurate quantitation and dynamic range for each lipid species. Supplemental Table 8a and 8b outline the standard cocktail for structural lipids for serum and brain. 4 mL chloroform:methanol (1:1, v/v) was added to each sample and the lipid extraction was performed as described^20,21^. Lipid extraction was automated using a customized sequence on a Hamilton Robotics STARlet system (Hamilton, Reno, NV) to meet the high-throughput requirements. Lipid extracts were dried under nitrogen and reconstituted in 68 µL chloroform:methanol (1:1, v/v). Samples were flushed with nitrogen and stored at − 20 °C. Samples were diluted 50 fold in isopropanol:methanol:acetonitrile:water (3:3:3:1, by volume) with 2 mM ammonium acetate in order to optimize ionization efficiency in positive and negative modes. Electrospray ionization-MS was performed on a TripleTOF 5600^+^ (SCIEX, Framingham, MA), coupled to a customized direct injection loop on an Ekspert microLC200 system (SCIEX) as described^7^. Lipid species with > 50% missing values were removed, with remaining missing values estimated (the half of the minimum positive values in the original data assumed to be the detection limit) and IQR filtered, normalized to the median, glog transformed, and autoscaled. Additional details about the ions used for quantitation can be found in Supplemental Table 9. One-way ANOVA and volcano plot visualization was performed using Metaboanalyst 4.0^22^.

### Mediator lipidomic anaylsis

A mixture of deuterium-labeled internal standards was added to aliquots of 100 µL serum, followed by 3 × volume of sample of cold methanol (MeOH). Samples were vortexed for 5 min and stored at − 20 °C overnight. Cold samples were centrifuged at 14,000 g at 4 °C for 10 min, and the supernatant was then transferred to a new tube and 3 mL of acidified H_2_O (pH 3.5) was added to each sample prior to C18 SPE columns (Thermo Pierce) and performed as described^23^. The methyl formate fractions were collected, dried under nitrogen, and reconstituted in 50 µL MeOH:H_2_O (1:1, v/v). Samples were transferred to 0.5 mL tubes and centrifuged at 20,000 g at 4 °C for 10 min. Thirty-five µL of supernatant were transferred to LC–MS vials for analysis using the BERG LC–MS/MS mediator lipidomics platform as described^24^.

### Proteomic analysis

Sixty-five µL of serum were delipidated using Lipisorb and then depleted using a multiple affinity removal spin cartridges Mouse-3 (Agilent Technologies). Low abundant proteins were collected in 100% Agilent Buffer A. Delipidated and depleted samples were used for determination of protein concentration using a Coomassie Bradford Protein Assay Kit (Thermo Pierce). Tissues were lysed using 7 M urea, 2 M thiourea, 1% Halt Protease and Phosphatase Inhibitor cocktail and 0.1% SDS, followed by sonication. After lysis, samples were centrifuged, and supernatant was used for proteomics analysis. The protein concentration was determined using Coomassie Bradford Protein Assay Kit.

Proteins were reduced in 10 mM Tris(2-carboxyethyl) Phosphine (TCEP) for 30 min at 55 °C and alkylated in 18.75 mM iodoacetamide for 30 min at room temperature in the dark. Proteins were precipitated overnight using acetone. Protein pellets were reconstituted in 200 mM tetraethylammonium bicarbonate (TEAB) and digested with trypsin at 1:40 (trypsin:protein) overnight at 37 °C. Peptides were then labeled with Tandem Mass Tag (TMT) 10-plex isobaric label reagent set (Thermo Pierce) using manufacturer’s protocol. Labeling reaction was quenched with 5% hydroxylamine for 15 min before being combined into each respective multi-plex (MP). Pooled samples were dried in a vacuum centrifuge followed by desalting using C-18 spin columns (Thermo Pierce). The eluate from C-18 was dried in a vacuum centrifuge and stored at − 20 °C until LC–MS/MS analysis.

LC–MS/MS analysis was performed using a Waters NanoAcquity 2D LC system coupled to a Thermo Q Exactive Plus MS. TMT-labeled samples were fractionated online into 12 basic reverse phase fractions. Each fraction was subjected to 90 min reverse phase separation. Data-dependent Top-15 acquisition method was used for MS analysis. Parameters used for Q-Exactive plus were full MS survey scans at 35,000 resolution, scan range of 400–1800 Thompsons (Th; Th = Da/z). MS/MS scans were collected at a resolution of 35,000 with a 1.2 Th isolation window. Only peptides with charge + 2, + 3, and + 4 were fragmented with a dynamic exclusion of 30 s.

Raw LC–MS/MS data were then processed using Proteome Discoverer v1.4 (Thermo) by searching a Swissport Mouse database (Swissprot 20 July 2016, 16,794 entities) using the following parameters for both MASCOT and Sequest search algorithms: tryptic peptides with at least six amino acids in length and up to two missed cleavage sites, precursor mass tolerance of 10 ppm, fragment mass tolerance of 0.02 Da; static modifications: cysteine carbamidomethylation, N-terminal TMT10-plex; and dynamic modifications: asparagine and glutamine deamindation, methionine oxidation, and lysine TMT10-plex. The mass spectrometry proteomics data have been deposited to the ProteomeXchange Consortium via the PRIDE partner repository with the dataset identifier PXD013146.

### Pathway analysis

Metabolome, lipidome, and proteome profiling results were processed separately using routine statistical tools. Pathway analysis was performed on Metabolomics data only using MetaboAnalyst package (https://www.metaboanalyst.ca/MetaboAnalyst/faces/home.xhtml) with a particular module—Statistical Analysis and Pathway Analysis. To document the most significant biological functions revealed by enrichment analysis and to identify novel relationships between metabolites detected and biological entities related to observed metabolic alterations, we performed a pathway analysis of all of the metabolite alterations detected. The pathway analysis module combines results from pathway enrichment analysis (MSEA) with the pathway topology analysis to help researchers identify the most relevant pathways involved in the conditions under study.

### Western blot

Protein was extracted from bi-lateral NAc punches from individual mice (AllPrep Qiagen Kit, Catalog: 80,204), suspended in 90 µL of 2 × Laemmli sample buffer, and 45 µL was run on a TGX stain free 4–20% gel (BioRad, Catalog: 5678093) at 150 V for 1.5 h. Protein was transferred to PVDF membrane using TransBlot Turbo packs (BioRad, Catalog: 1704157) for 14 min. The PVDF membrane was blocked in 3% bovine serum albumin (BSA) in TBST for 1 h, followed by 4 °C overnight incubation of the RPRD2 primary antibody (Abcam, Ab10363, 1:2000) suspended in 3% BSA blocking buffer. The following morning, the membrane was washed five times in TBST with 5 min between each wash and then incubated in secondary antibody (Abcam, Ab205723, 1:10,000) in 5% skim milk in TBST for 1 h. The membrane was again washed five times in TBST, exposed to Clarity ECL substrate (BioRad, Catalog: 1705060), and developed in the ChemiDoc MP instrument (BioRad, Catalog: 12,003,154). In Image Lab software (BioRad, Catalog: 1709690), RPRD2 band intensity was quantified and normalized to total protein in the lane. Total protein was visualized via brief UV illumination of the stain free gel on the ChemiDoc MP instrument. On the resulting image, total lane pixel intensity was quantified in Image Lab, enabling total protein quantification according to established methods^25^. Data were expressed as a ratio of susceptible mice.

### Statistical corrections

In the main text, significance is reported at uncorrected p < 0.05 for broad pattern identification. However, 5% FDR corrections were performed and are reported in the supplemental tables. Features that survive FDR correction are emphasized within the text and figures.

## Results

### Widespread molecular changes are induced by chronic social defeat stress

To resolve the diverse molecular changes that occur within the brain and serum of mice in response to chronic social stress, we exposed C57BL/6J mice to ten days of CSDS, and 24 h after the final defeat we performed a social interaction (SI) test to identify susceptible or resilient mice (*F*_2,117_ = 32.82; ***p < 0.0001; one-way ANOVA followed by Bonferroni post-test) (Fig. 1A,B). Previous work has established that the SI test is highly correlated with many other behavioral, cellular, and molecular outcomes after CSDS^2,3,5,9^. We then performed metabolomic, lipidomic, and proteomic profiling in parallel on pooled samples of serum and of vHipp, NAc, and mPFC of susceptible, resilient, and undefeated control mice to capture the full spectrum of molecular changes in blood and brain that differentiate these populations (Fig. 1C,D).

In serum, metabolomic analysis identified 61 affected metabolites by one-way ANOVA comparing susceptible, resilient, and control groups. The identities of these metabolites along with post-hoc analyses and FDR correction are presented in Supplemental Table 1A. A heatmap of the top 25 affected metabolites with supervised clustering by group shows differences in peripheral metabolite levels localized to each group (Fig. 1E). Pathway analysis identified many of the affected metabolites as important in the biological functions of nucleotide metabolism as well as energy production and antioxidant function (Fig. 1F). Lipidomic analysis identified 1179, 1177, and 1184 unique lipid species across 21 lipid classes for the control, susceptible, and resilient groups, respectively, with no significant group differences in the number of identifiable lipid species. However, we detected significant changes in oxidized lipid mediators, as well as changes in the concentration of 57 structural lipid species; for example, total serum levels of all phosphatidic acid (PA) species were significantly increased in resilient animals relative to susceptible (*F*_2,58_ = 3.80; *p < 0.05; one-way ANOVA followed by Fisher’s LSD) (Fig. 1G, list of structural lipid species in Supp. Table 1B; list of 9 oxidized/mediator lipids affected in Supp. Table 1C). Lastly, proteomic analysis detected 450 unique proteins in serum, with a total of 17 identified as significantly different from control animals (Supp. Table 11D). Three proteins were significantly decreased exclusively in the serum of susceptible animals: kallikrein B1 (Klkb1), murinoglobulin-1 (Mug1), and thyroid receptor-interacting protein 11 (Trip11) (Fig. 1H).

**Figure 2:**
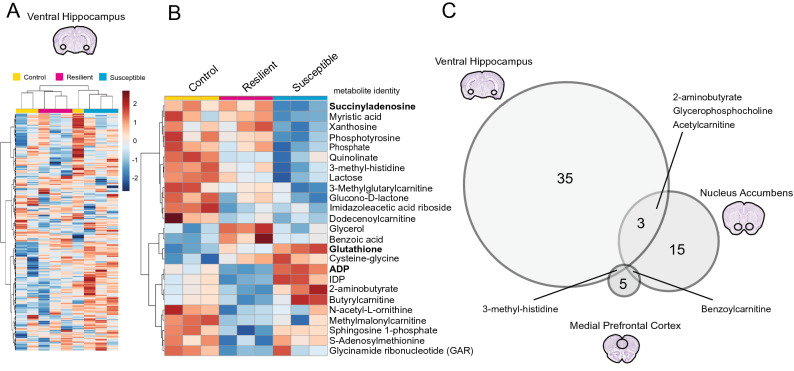
Metabolites are differentially affected by chronic stress in each brain region. (**A**) Ventral hippocampus (vHipp) samples show robust metabolic changes after CSDS. Unsupervised clustering of metabolite heatmaps largely aggregate by group, revealing substantial differences in metabolic composition that differentiate control, resilient, and susceptible animals. (**B**) A supervised heatmap of the top 25 significantly affected metabolites reveal dramatic differences in metabolite concentrations in each group; metabolites that survive FDR correction are in bold. (**C**) 39, 19, and 7 metabolites were impacted in the vHipp, nucleus accumbens (NAc), and medial prefrontal cortex (mPFC), respectively with overlapping metabolites highlighted. Heatmaps in A and B were generated with MetaboAnalyst 4.0 (https://www.metaboanalyst.ca/).

Stress responses are mediated through the functions of several brain regions^26^. To investigate how chronic stress impacts the molecular composition of key brain regions, we performed multi-OMIC analyses on vHipp, NAc, and mPFC tissues. With metabolomic analysis in the vHipp, an unsupervised heatmap of all detected metabolites clustered according to group, indicating group differences in general metabolite composition (Fig. 2A). We identified 39 altered metabolites (identities in Supp. Table 2A). A supervised heatmap of the top 25 affected metabolites shows concentration differences between groups, with many affected metabolites being purine nucleotides, carnitine donors and modified fatty acids involved in beta oxidation, and antioxidants (Fig. 2B). The same metabolomic detection analyses performed in the NAc revealed 19 altered metabolites (identities in Supp. Table 2B), and only seven in the mPFC (identities in Supp. Table 2C), revealing a different metabolic impact of chronic stress by brain region (Fig. 2C). Further, relatively few of the same metabolites were impacted across brain regions, with no specific metabolite affected in all three brain regions (Fig. 2C).

To understand whether the effect of CSDS on these various metabolites was associated with susceptibility or resilience by brain region, we performed untargeted metabolomic profiling in parallel to our targeted analyses. This separate analysis detected many of the same or similar molecules that were identified de novo (Supp. Table 3). Comparing CSDS-exposed mice to undefeated controls, untargeted metabolomics identified 34 metabolites significantly altered in the vHipp, and 11 metabolites significantly altered in the NAc (Supp. Figure 1), again revealing a different metabolic response to stress by brain region. In vHipp the regulation of identified molecules occurred primarily in susceptible animals, whereas in the NAc alterations occurred entirely in resilient animals. Thus, metabolic impact in the NAc is associated with resilience, with metabolic impact in the vHipp associated with susceptibility to chronic stress.

With lipidomic analysis, we observed 36 altered lipid species in the vHipp with multiple species of long chain phosphatidylethanolamine (PE), a major constituent of the myelin sheath, being decreased in susceptible animals (Supp. Table 4A). We identified 54 altered lipid species in the NAc (Supp. Table 4B), and 28 lipid species in mPFC (Supp. Table 4C). With proteomic analysis, we observed 51 altered proteins in the vHipp (Supp. Table 5A), 53 in the NAc (Supp. Table 5B), and 71 in the mPFC (Supp. Table 5C). To interrogate the reliability of our findings, we performed western blot analysis in a separate CSDS cohort for the only protein affected in all three brain regions and the top affected protein in the NAc, RPRD2 (regulation of nuclear pre-mRNA domain containing 2), which plays an important role in gene transcription and was predicted to be elevated in resilient animals relative to susceptible in the NAc. Indeed, we validated this proteomic deduction, which supports the reliability of our analyses (Supp. Figure 2). Further, we performed Kyoto Encyclopedia of Genes and Genomes (KEGG) pathway analysis^27,28^ of the proteome alterations for each brain region (Supp. Table 5D-F).

### Imipramine treatment regulates key molecules in the brain and periphery

**Figure 3:**
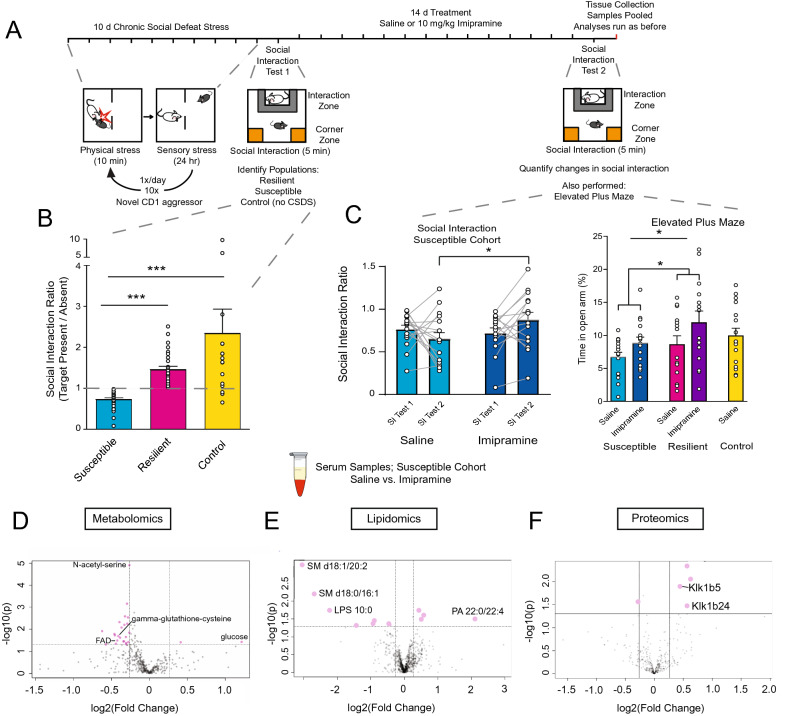
Chronic imipramine treatment improves susceptible behaviors and correlated peripheral molecular profiles. (**A**) Graphical illustration of workflow for CSDS, treatment schedule, and behavioral testing. Serum, vHipp, NAc, and mPFC were again harvested, pooled by SI ratio, and analyzed as before (see Fig. 1A). (**B**) SI test 1 data from all mice used to determine susceptible, resilient, and control populations. Animals were pooled into future treatment groups based on equalized SI ratios. Susceptible (n = 33), Resilient (n = 28) and Control (n = 16) (*F*_2,74_ = 18.61; ***p < 0.0001; one-way ANOVA followed by Bonferroni post-test). (**C**) Post-treatment behavioral data including SI and elevated plus maze (EPM). Left: Treatment-induced changes to SI ratios in susceptible population comparing SI test 1 and SI test 2 (n = 14–16; *F*_1,28_ = 4.423; * = *p* < 0.05 by repeated measures two-way ANOVA followed by Bonferroni post-test). Right: EPM test quantifying anxiety-like behaviors in all groups. Resilient animals spend more time exploring the open arms than susceptible animals (*F*_1,56_ = 4.19; main effect of group * = *p* < 0.05 by two-way ANOVA), and treatment significantly improves anxiety-like behaviors (*F*_2,56_ = 4.93; main effect of treatment * = *p* < 0.05 by two-way ANOVA). (**D**) Metabolomic analysis of serum from imipramine-treated susceptible cohort. Volcano-plot highlights 25 important features. (**E**) Volcano plot of the 11 affected lipids. (**F**) Volcano plot reveals five affected proteins. n = 8 samples per group for panels D-F. Volcano plots in D-F were generated with MetaboAnalyst 4.0 (https://www.metaboanalyst.ca/).

To investigate the molecular effects of imipramine treatment, we again stratified C57BL/6 J mice into resilient and susceptible populations after CSDS (*F*_2,74_ = 18.61; ***p < 0.0001; one-way ANOVA followed by Bonferroni post-test) (Fig. 3A,B) and divided animals into their future treatment groups by equalizing SI ratios. Susceptible, resilient, and control mice were treated twice daily for 14 days with an IP injection of either saline or imipramine (10 mg/kg) (Fig. 3A). Twenty-four hr after the last injection, all mice were examined in a second social interaction test, as well as an elevated plus maze test, to determine the effect of imipramine exposure.

As expected^5^, chronic imipramine administration improved social interaction deficits seen in susceptible mice. We compared the SI ratio from test 1 (before treatment) and SI test 2 (after treatment), and while saline treatment did not improve sociability, there was a significant improvement in SI ratio in imipramine-treated susceptible mice (n = 14–16; *F*_1,28_ = 4.423; * = *p* < 0.05 by repeated measures two-way ANOVA followed by Bonferroni post-test) (Fig. 3C).

We next performed the elevated plus maze assay. We observed a significant difference in time spent on the open arm between susceptible and resilient groups, with the former group spending less time in the open arm, indicating that group phenotypes persisted throughout the treatment window (*F*_1,56_ = 4.19; main effect of group * = *p* < 0.05 by two-way ANOVA). We also observed a significant treatment effect in both susceptible and resilient groups with imipramine treatment increasing the amount of time spent exploring the open arm (*F*_2,56_ = 4.93; main effect of treatment * = *p* < 0.05 by two-way ANOVA). (Fig. 3C). Collectively, these data indicate that imipramine improves behavioral abnormalities induced by CSDS. To understand the molecular pathways affected by imipramine exposure in chronically-stressed mice, we performed multi-OMIC profiling on pooled samples of serum and of vHipp, NAc, and mPFC of all mice.

In the serum of susceptible animals treated with saline or imipramine, metabolomic analysis identified 25 significantly affected metabolites (Fig. 3D, identities in Supp. Table 6A). Notably, imipramine treatment reduces the levels of many metabolites, with many involved in previously identified functions including the antioxidant precursor gamma-glutathione-cysteine and the coenzyme flavin adenine dinucleotide (FAD) which participates in many metabolic functions including the TCA cycle.

With lipidomic analysis, we observed that imipramine treatment significantly affected the serum concentrations of 11 lipids (Fig. 3E, identities in Supp. Table 6B): sphingomyelin and lysophosphatidylserine species were decreased, and there was an increase in a PA species, which is consistent with the earlier-observed increase in PA species specifically in resilient animals.

Lastly, with proteomic analysis, we identified five significantly affected proteins (Fig. 3F, identities in Supp. Table 6F). Kallikrein 1-related peptidase b5 (Klk1b5) and Kallikrein 1-related peptidase b24 (Klk1b24) were each increased by imipramine treatment. This is consistent with our earlier observation of decreases in Klkb1 in the serum of susceptible animals (Fig. 1H), suggesting that imipramine may act to increase Klkb1-related functions. This observation highlights the potentially important role for peripheral protease activity in contributing to stress responses.

**Figure 4:**
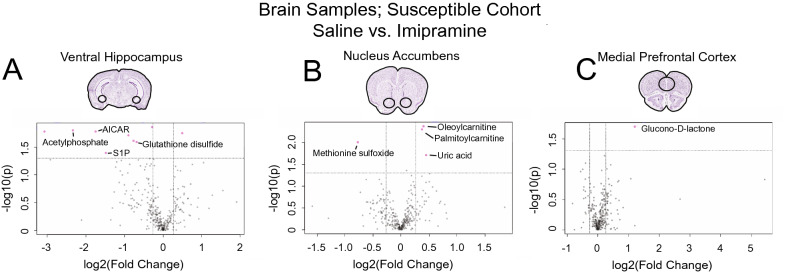
Imipramine-induced molecular adaptations by brain region within susceptible animals. (**A**) Volcano plot of nine changing metabolites identified in the vHipp. n = 3 samples per group. (**B**) Volcano plot of four changing metabolites identified in the NAc. n = 5 samples per group. (**C**) Volcano plot of one changing metabolite identified in the mPFC. n = 5 samples per group. Identities and statistics for all metabolites can be found in Supplemental Table 7A-C. Volcano plots in A-C were generated with MetaboAnalyst 4.0 (https://www.metaboanalyst.ca/).

We then performed metabolomic analysis in each brain region. In the vHipp, as in serum, we observed primarily a down-regulation of key metabolites (including AICAR involved in de novo purine synthesis, the antioxidant glutathione disulfide, S1P, and acetylphosphate), with nine metabolites significantly affected (Fig. 4A, list in Supp. Table 7A). A heatmap of the top 25 affected metabolites clusters by treatment, revealing how imipramine treatment affects the vHipp and acts to diminish the levels of metabolites that were previously observed to be elevated in the vHipp of susceptible animals (Supp. Figure 3). In the NAc, four metabolites are affected (Fig. 4B, list in Supp. Table 7B). Lastly, only one metabolite was significantly affected in the mPFC (Fig. 4C, list in Supp. Table 7C). These data mirror the observations of Fig. 2, in that the brain areas that were most affected metabolically by stress are also most affected by imipramine treatment.

### Metabolic network mapping

**Figure 5:**
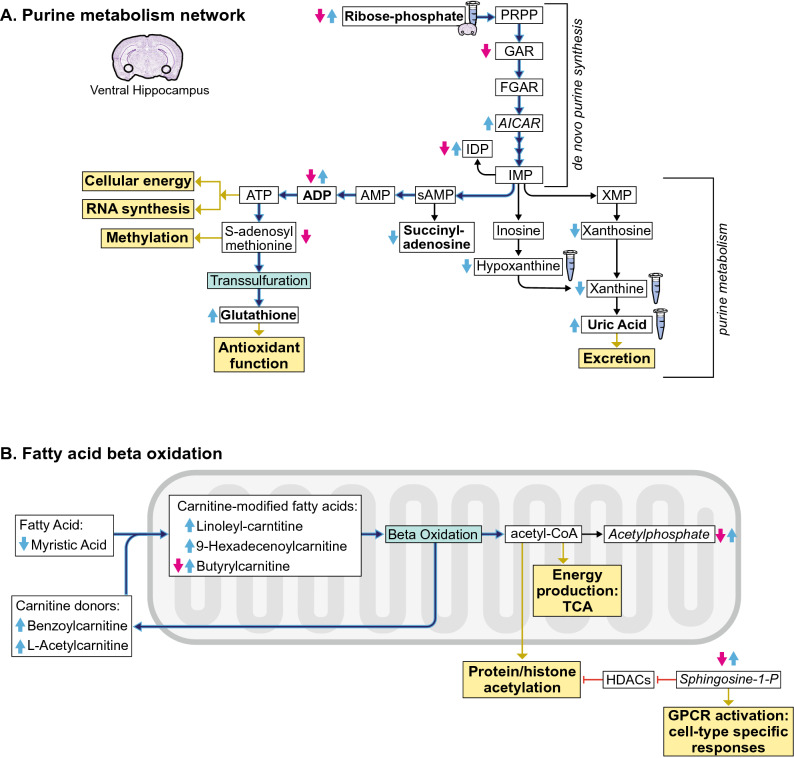
Ventral hippocampus metabolic networks differentially affected in susceptibility vs resilience. Red and blue arrows indicate regulation of metabolite levels observed across our datasets in resilient and susceptible animals, respectively. The test tube icon represents an alteration observed in serum samples, as these molecules are excreted in urine. The hyperactive pathway through these networks experienced by susceptible animals is highlighted in blue. Metabolite relationships were derived from KEGG database and the IUBMB-Sigma-Nicholson Metabolic Pathways Chart. Solid black arrows represent direct metabolic reactions. Yellow boxes represent basic cellular functions affected by key metabolites. Molecules that survived FDR correction are in bold. Molecules affected by imipramine treatment are italicized. PRPP, phosphoribosyl pyrophosphate; GAR, glycinamide ribonucleotide; FGAR, 5′-Phosphoribosyl-N-formylglycineamide; AICAR, 5-Aminoimidazole-4-carboxamide ribonucleotide; IMP, inosine monophosphate; IDP, inosine diphosphate; XMP, xanthosine monophosphate; sAMP, succinyl adenosine monophosphate; AMP, adenosine monophosphate; ADP, adenosine diphosphate; ATP, adenosine triphosphate.

We utilized the metabolomics data in serum and brain to construct the net effect of CSDS and imipramine treatment on metabolic pathways. In the vHipp, we observed consistent changes in many of the metabolites within the de novo purine synthesis pathway with resilient animals showing decreases and susceptible animals showing increases in these metabolites (Fig. 5A). Many purine products are decreased in susceptible animals. ADP, a precursor of ATP, is significantly elevated in susceptible and decreased in resilient animals. A key product of ATP, s-adenosyl methionine (SAM-e) is diminished in the vHipp of resilient animals. SAM-e is a major methyl donor used for protein and lipid methylation, and differences in its bioavailability within the vHipp could have sweeping consequences on cellular functions.

In the vHipp, beyond the purine network, susceptible and resilient animals show opposing effects in fatty acid beta oxidation pathways. Susceptible animals show hyperfunction of the fatty acid beta oxidation cycle (Fig. 5B). Beta oxidation is the process through which fatty acids are internalized in the mitochondria via carnitine modification and consumed to yield acetyl-CoA^29^. These susceptible-specific effects in glycolysis and beta oxidation converge in the accumulation of acetyl-CoA, further implicating hyper-energetics in the vHipp of susceptible animals. Also, acetyl-CoA is a source for protein acetylation, including histones^30^. This potential regulation of histone acetylation levels is consistent with a literature in both mice and rats describing changes in hippocampal histone acetylation in response to stress and antidepressant exposure^5,31–33^.

We were unable to resolve significant differences in ATP or acetyl-CoA themselves, likely due to the fact that these metabolites are consumed rapidly and utilized for numerous cellular functions. However, resolving the full metabolic network, including precursor and product molecules, serves to reveal metabolic demands. Taken together, these analyses demonstrate that the brain-region specific cellular functions of energy production and the global post-translational modifications of methylation and acetylation are likely hyperactive in the vHipp of susceptible animals. This illuminates that individual differences in these cellular functions by brain-region may underlie the divergence in stress responses.

## Discussion

Here, we generated a comprehensive dataset that quantifies the protein, lipid, and metabolite changes that occur across the brain and serum of chronically-stressed and imipramine-treated mice. This is a uniquely-detailed dataset, from which many fundamental discoveries relating to the biological mechanisms underlying stress responses and antidepressant action can be derived. Analyzing these data, we discovered that chronic stress affects the molecular pathways of purine metabolism, beta oxidation, and antioxidant function and that such changes associate with phenotypic differences in behavioral responses to stress, depending on the brain region in which the adaptations occur.

We observed that the three brain regions studied display marked differences in metabolic responses to chronic stress: the vHipp is the most robustly affected, followed closely by the NAc, with the mPFC not as affected by chronic stress by these metrics. By contrast, the mPFC of mice and humans is affected dramatically by chronic stress and MDD, respectively, at the transcriptional level^1,34^, raising the interesting possibility that different brain regions may undergo different modes of biological regulation under these conditions. Similarly, we observed minimal coordinated impact to proteins involved in purine metabolism or beta oxidation in our proteomic analysis in the brains of stressed mice which is in direct contrast to our metabolomic findings wherein the metabolites of these networks were robustly affected. This raises the possibility that, depending on the method of molecular analysis, considerably different conclusions may be reached and argues in favor of a multi-OMIC approach as employed in the present study.

Considering that the vHipp is instrumental in processing both depression- and anxiety-like behavior^35,36^, it is not surprising that this region is so heavily affected by chronic stress. Our findings further illuminate that a major difference between stress-resilient and -susceptible mice is the activity of specific molecular pathways within this brain region (Fig. 5). Susceptibility may manifest from metabolic processing to support the hyperactivity of key cellular functions, such as ATP synthesis and availability of molecular reactants for protein post-translational modifications. In support of this notion, treating stressed animals with imipramine down-regulated these molecular substrates in the vHipp and improved CSDS-induced behavioral abnormalities. Resilience is associated with the absence of molecular reactants within the vHipp. In contrast to vHipp, hyperactivity of largely the same molecular pathways within the NAc, a brain region instrumental in processing reward, is correlated with resilience. Therefore, we can conclude that the outlined molecular pathways are sensitive to chronic stress, but that the brain region in which altered function occurs differentiates the animal’s stress susceptibility vs resilience.

Many carnitines, implicated in human depression and animal stress models^37^, are central to the function of beta oxidation and were upregulated in the NAc of susceptible animals treated with imipramine (Supp. Table 7B). The availability of cellular energy would affect the functioning of these brain regions. Furthermore, reduced neuronal activity in the vHipp is observed in animals resilient to CSDS, with its optogenetic activation promoting susceptibility^7^. These observations suggest that imipramine might induce its therapeutic effect, in part, by regulating brain-region specific energy production, and highlights amenable molecular pathways to target with novel antidepressants.

To extrapolate which cell types within the analyzed brain regions might be most impacted, we investigated the brain region-specific profiles of neurotransmitters in our datasets. Within the vHipp, resilient animals display reductions in dihydroxyphenylalanine (L-DOPA; Supp. Table 2A), a precursor of dopamine, suggesting reduced activity of dopaminergic afferents in the vHipp of resilient animals. Further, γ-aminobutyric acid (GABA) levels are decreased in the vHipp of susceptible animals, indicating a possible reduction in local inhibitory GABAergic tone. These data are consistent with our findings across multiple levels of analysis that hyperfunction of pyramidal projection neurons of the vHipp is associated with susceptibility and hypofunction is associated with resilience^7^.

Lipids present another promising class of molecular targets for novel antidepressants and are increasingly appreciated as contributing to MDD and related disorders^38^. We observe carnitines involved in fatty acid beta oxidation as the most dramatically and consistently affected class of molecule across brain regions. In the vHipp, long chain species of PE were depleted in susceptible animals, a potential indication of demyelination as well as of reactive oxygen species. There is evidence that PE methylation is the primary consumer of SAM-e, and in instances of PE depletion, histone proteins replace PE as the primary methyl sinks^39^. Considering that resilient animals have reductions in SAM-e levels in vHipp, and susceptible animals have depletions in PE lipids, this evidence converges to suggest that methylation, and specifically histone methylation, might be oppositely regulated in this brain region of susceptible vs resilient mice, consistent with a role for this histone modification in stress responses^40^. Also, considering reports that SAM-e may show some antidepressant efficacy^41^, it is conceivable that any therapeutic effect could be mediated via regulation of methylation within the vHipp.

In serum, we detected many stress-induced alterations in levels of lipid species. In resilient animals, several PA species were elevated, and the top up-regulated lipid in response to antidepressant treatment was a long-chain PA (Fig. 3E). PA is involved in phospholipid biosynthesis, membrane remodeling, and second messenger signaling, and regulates immune response by stimulating macrophage function^42^. Lysophopshatylserine (LPS) positively regulates inflammatory responses through activation of mast cells^43^ and was down-regulated in response to antidepressant treatment. The combined effects for PA and LPS species implicate peripheral inflammation and the immune system as highly sensitive to chronic stress and amenable to regulation with antidepressant treatment. In support of this notion, there is growing evidence that inflammation and the peripheral immune system helps drive behavioral responses to stress^44^. Such responses are unrelated to the mild physical injury associated with CSDS^44^. Furthermore, we observed increases in sphingomyelin (SM) medium chain lipids (*F*_2,58_ = 5.21; *p* < 0.05; Fisher’s LSD) and ceramide monounsaturated lipids (*F*_2,58_ = 4.48; *p* < 0.05; Fisher’s LSD) specifically in the serum of susceptible animals with decreased levels of a SM species upon imipramine treatment. Plasma ceramide levels are elevated in individuals with MDD^45^ and many antidepressants, including imipramine, inhibit the functions of acid sphingomyelinase^46^, which catalyzes the degradation of SM to ceramide. These data collectively highlight a possible role for peripheral lipids as markers and mediators of MDD.

Lastly, this dataset offers a valuable resource in identifying circulating peripheral markers of stress susceptibility vs resilience. High levels of the serum metabolites flavin adenine dinucleotide (FAD) and uric acid, among others, are associated with susceptibility, and low levels of bilirubin are associated with resilience. Interestingly, uric acid and bilirubin have been associated with MDD in humans^47,48^, indicating the translational potential of our data. While the three brain regions studied here shared ~ 15% overlap in metabolite impact with our serum data, no one metabolite was uniformly disrupted across all tissues. However, when looking at a class of molecules like carnitines and carnitine modified-fatty acids, serum changes are a useful marker for brain alterations. Intriguingly, affected peripheral peptides were largely the same in susceptible and resilient animals, which limits their utility as biomarkers. Thus, detection of a molecular profile that spans the described classes of molecules may improve reliability and is an important area of future efforts.

Recently, as alluded to earlier, circulating levels of acetylcarnitine, which is an epigenetic regulator and participates in beta oxidation, was reported to be diminished in individuals with MDD^37^. While circulating levels did not reach statistical threshold in our dataset, we did observe acetylcarnitine as significantly increased in the vHipp of susceptible animals and increased in the NAc of resilient animals, reinforcing our findings on the opposing and brain region-specific contributions to CSDS-induced abnormalities.

It is important to note that a limitation of the present study is its focus on male mice. Given the increased prevalence of MDD in females^49^, and the very different transcriptional profiles observed across brain regions between MDD males and MDD females^1^, it is possible that the molecular pathways identified herein may not be similarly impacted in females. Moreover, considering there is emerging evidence that there are sex differences in antidepressant treatment efficacy^50^, further research is required to investigate the molecular impact of chronic stress and antidepressant treatment in females. We elected to not treat our control mice with imipramine since early evidence with imipramine suggested that it does not produce effects on mood in control humans and only produces side effects^5^ (see Supplementary Note #2)*.* Further, the 14-day imipramine treatment meant that mice in the two CSDS experiments were euthanized at different times relative to the first SI test. This limited our ability to directly compare across experiments given the observation of a longitudinal effect on the expression of some CSDS-related behaviors^51^. It would be interesting in future studies to examine multi-OMIC endpoints as a function of time after the defeat stress. Another limitation is the tissue pooling approach required for parallel OMIC analyses on each micro-dissected brain region. We chose to pool samples since bilateral brain regions of individual mice can total less than 2 mg of tissue (i.e., vHipp), which was not sufficient for performing metabolomics, lipidomics, and proteomics in parallel. Since tissues from multiple mice were pooled, we are unable to correlate molecular profiles with individual animal behavior. Pooling tissue is an approach used by other groups to perform parallel analyses on limited starting tissues^52^. As the sensitivity of OMIC analyses improves, studies of tissues from individual animals, or even specific cell-types, should be performed to overcome the limitations of the present work.

Multiple earlier studies investigated metabolomic and proteomic changes in rodent stress models. In male mice and hamsters subjected to acute social defeat stress, stress resistant animals were reported to have increases in metabolites that protect against oxidative stress within the NAc and mPFC^53^. In the hippocampus of rats subjected to physical or psychological stressors, lactic acid was one of nine metabolites altered in all stress contexts, revealing the universal impact of chronic stress on glycolysis and mitochondrial function of this brain region^54^. And in rats subjected to chronic unpredictable stress, disturbances in purine metabolism were identified in the olfactory bulb^55^, hinting that purine metabolism may be sensitive to stress in brain regions that were not investigated in the present study.

In summary, our datasets offer new insights into the molecular mechanisms of MDD and its treatment, as we identified a range of molecular changes induced in serum and specific brain regions after CSDS in susceptible vs resilient mice and in response to chronic imipramine administration. Further analysis of these datasets may guide efforts to construct reliable peripheral markers of MDD and its treatment and uncover novel molecular pathways for therapeutic discovery efforts.

## Supplementary information

Supplementary information 1.

Supplementary information 2.

Supplementary information 3.

Supplementary information 4.

Supplementary information 5.

Supplementary information 6.

Supplementary information 7.

Supplementary information 8.

Supplementary information 9.

Supplementary information 10.
