# Supplementary material for: Chronic stress and antidepressant treatment alter purine metabolism and beta oxidation within mouse brain and serum
Source: Sci Rep. 2020 Oct 22;10:18134. doi: 10.1038/s41598-020-75114-5 (PMC7582177; doi:10.1038/s41598-020-75114-5)
Supplement: Supplementary file 10 — Supplementary information 10. [file 41598_2020_75114_MOESM10_ESM.docx]

**SUPPLEMENT 1**

TITLE

Chronic stress and antidepressant treatment alter purine metabolism and beta oxidation within mouse brain and serum

SHORT TITLE

Multi-OMIC analyses of stressed mice

Peter J. Hamilton^1,2^, Emily Y. Chen^3^, Vladimir Tolstikov^3^, Catherine J. Peña^1^, Joseph A. Picone^2^, Punit Shah^3^, Kiki Panagopoulos^3^, Ana N. Strat^1^, Deena M. Walker^1^, Zachary S. Lorsch^1^, Hannah L. Robinson^2^, Nicholas L. Mervosh^1^, Drew D. Kiraly^1^, Rangaprasad Sarangarajan^3^, Niven R. Narain^3^, Michael A. Kiebish^3^, Eric J. Nestler^1^

^1^Nash Family Department of Neuroscience and Friedman Brain Institute, Icahn School of Medicine at Mount Sinai, One Gustave L Levy Place, New York, NY 10029 ^2^Department of Anatomy & Neurobiology, Virginia Commonwealth University, Richmond, VA, 23298 ^3^BERG LLC, 500 Old Connecticut Path, Framingham, MA 01701


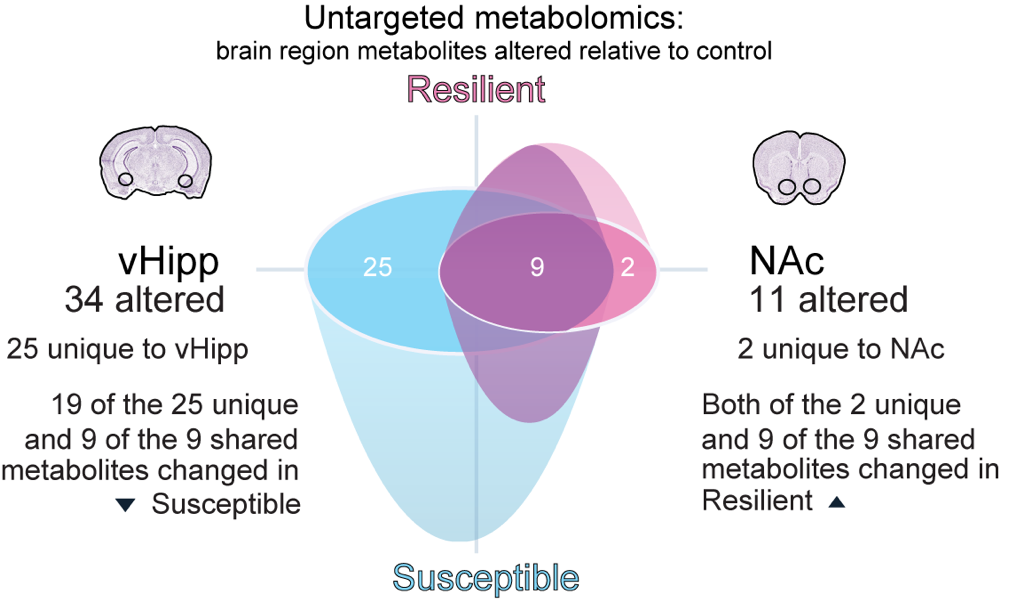


**SUPPLEMENTAL FIGURE 1**. **Metabolic impact is differentially associated with susceptibility or resilience.** Separate untargeted metabolomic analysis comparing undefeated controls to CSDS exposed mice demonstrates impact to the vHipp with 34 significantly altered metabolites. In the NAc, 11 affected metabolites are detected, nine of which are affected in both the vHipp and the NAc. However, metabolic changes in the NAc are associated with resilience to CSDS, while metabolic changes in the vHipp are associated with susceptibility to CSDS, since all metabolites in the NAc are solely altered in resilient animals, whereas a majority of the metabolites in the vHipp are altered in susceptible animals.


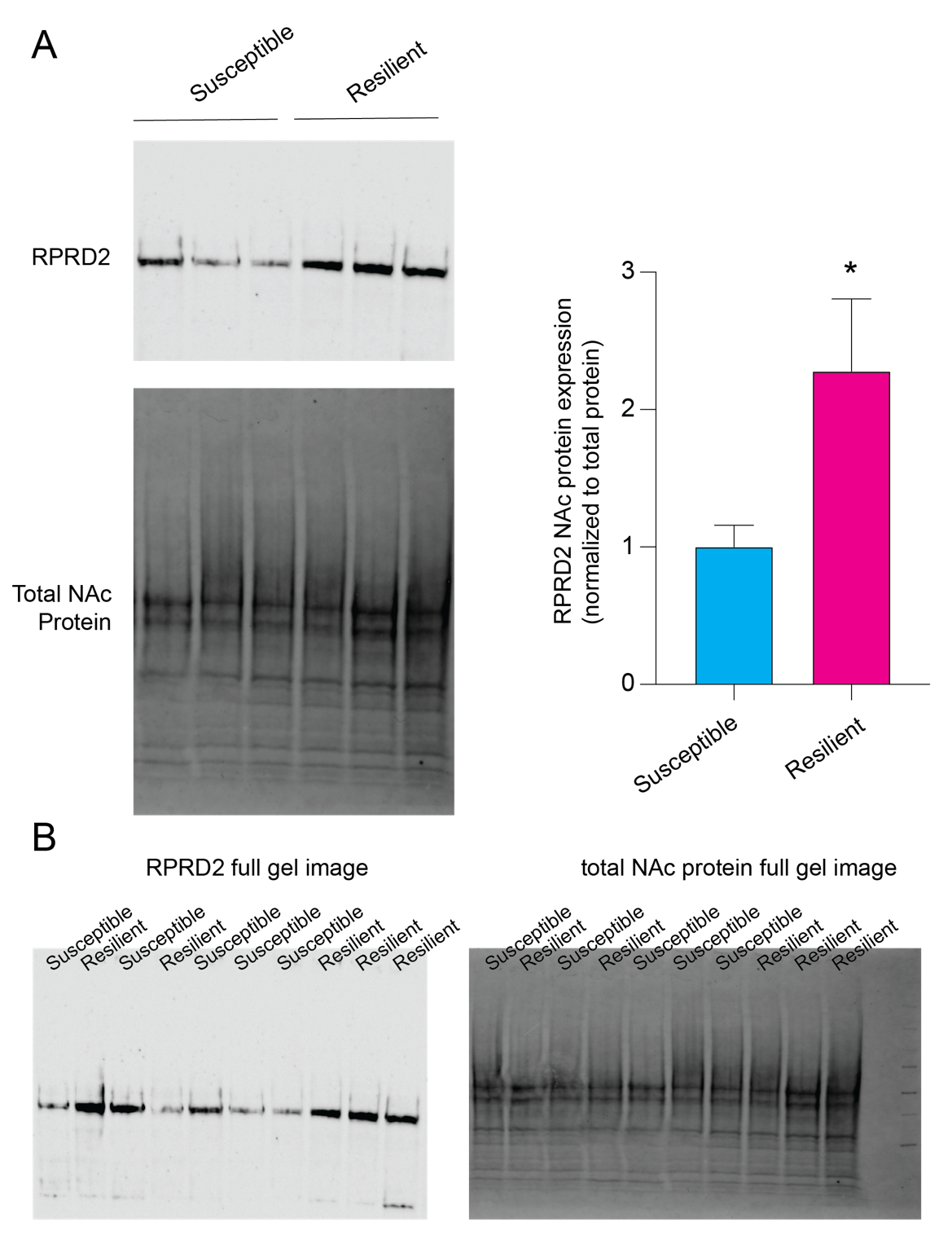


**SUPPLEMENTAL FIGURE 2**. **Western blot validation of brain proteomic analysis.** (**A**) In an independent CSDS cohort, the NAc of five susceptible and five resilient mice were dissected and probed for the top affected proteomic identified protein, RPRD2. Left, Top: Representative immunoblots for RPRD2 from three mice from each group. Bottom: Total NAc protein. Right: RPRD2 band intensity was quantified, normalized to total protein lane intensity, and expressed as a ratio of susceptible samples (n = 5/group; *p* < 0.05; two-tailed *t* test). (**B**) Full gel images

**SUPPLEMENTAL FIGURE 3**. **Imipramine treatment impacts the metabolic profile of the susceptible animal’s ventral hippocampus.** An unsupervised heatmap of the top 25 affected metabolites within susceptible animal’s vHipp shows divergent effects to nearly all metabolites and distinct clustering by treatment. Heatmap generated with MetaboAnalyst 4.0 (https://www.metaboanalyst.ca/).
